# A compositional analysis of systemic risk in European financial institutions

**DOI:** 10.1007/s10436-023-00427-0

**Published:** 2023-04-18

**Authors:** Anna Maria Fiori, Francesco Porro

**Affiliations:** 1 Dipartimento di Statistica e Metodi Quantitativi, Università degli Studi di Milano-Bicocca, Via Bicocca degli Arcimboldi 8, 20126 Milan, Italy; 2 Dipartimento di Matematica, Università degli Studi di Genova, Via Dodecaneso 35, 16146 Genoa, Italy

**Keywords:** Systemic risk share, SRISK, Compositional Data (CoDa), Aitchison geometry, Logratio coordinates, C10, C40

## Abstract

Systemic risk is a complex and multifaceted phenomenon that needs to be addressed from different perspectives. In this work we propose a Compositional Data (CoDa) approach to analyze the distribution of relative contributions to systemic risk associated with major European countries during the period 2008–2021. We represent systemic risk measures corresponding to those countries as percentage shares, or parts, of a compositional dataset and we perform a multivariate statistical analysis using specific CoDa procedures. The proposed approach sheds new light on some variability patterns and cross-country relationships that appear to be linked to the composition of systemic risk parts in the system.

## Introduction

The rising globalization of financial services and the consequences of the 2007–2009 financial crisis have prompted a vigorous debate on banking and insurance regulation in view of ensuring stability to the whole financial system. This debate has centered on the concept of *systemic risk*, which is defined as “a risk of disruption to financial services that is caused by an impairment of all or parts of the financial system and that has the potential to cause serious negative consequences for the real economy” (International Monetary Fund 2009). Nowadays, detecting systemic risk and identifying appropriate measures for its mitigation are among the priorities of macroprudential policies, which are intended to make the financial sector more resilient and to encourage the adoption of a system-wide perspective in financial regulation. In particular, the recent reforms of the Basel Accords include additional supervisory requirements for Global Systemically Important Financial Institutions (the so-called G-SIFIs), which are subject to capital surcharges proportionate to the risks they pose to the financial system and to the economy at large (Basel Committee on Banking Supervision 2018).

The case of European institutions has attracted considerable attention from both the financial markets and the academic community. As pointed out by Engle et al. (2014), a financial crisis in Europe could be triggered by several events, either at a worldwide level (such as the subprime crisis), or in a specific region (e.g., the sovereign debt crisis), or else on a national scale (e.g., the Greek debt crisis). Black et al. (2016) have shown that systemic risk in the European banking system reached a peak at the end of 2011, caused not only by changes in investors’ sentiment but also (and perhaps more importantly) by a real increase in the insolvency risk of European banks. Furthermore, their study distinguishes the role of German and UK banks from that of Italian and Spanish ones. While the former have been historically exposed to US subprime mortgage securities and thus directly affected by the collapse of Lehman, the latter were minor contributors of systemic risk prior to the Global Financial Crisis (GFC) but rapidly rose in systemic importance as fear of contagion spread from Greece to other European countries. Focusing on firms listed in the EuroStoxx 50 Index, Petrella et al. (2018) find that, between 2008 and 2017, the systemic risk contribution of France dominated all other countries, followed by Germany, Italy, and Spain. Stolbov and Shchepeleva (2018) investigate salient features of European countries with a view on identifying common patterns and real effects of systemic risk on the European economy. The frailty of the European financial and banking system is specifically addressed in Billio et al. (2016), with a proposal for the construction of an entropy-based early warning indicator of systemic risk. Using a decomposition of the Gini inequality index, Fiori and Porro (2020) detect not only a relevant contribution of Europe to the worldwide concentration of systemic risk, but also a considerable disparity among different institutions within the European continent. As a result of the ongoing regulatory efforts and the vast body of research conducted in the last decade, supervisory authorities and policy makers are now in a position to monitor the evolution of systemic risk with a variety of instruments and consolidated methodologies. Nonetheless, it is widely recognized that systemic risk is a multifaceted concept, which needs to be addressed from manifold perspectives.

Both the regulators and the academic community have usually quantified the “size” of systemic risk using either monetary units or scores: popular indicators expressed in monetary units include, among others, SRISK and CoVaR-based measures (see, e.g., Stolbov and Shchepeleva 2018, for a comparative analysis); conversely, a prominent example of score-based methodology is the one adopted by the Basel Committee on Banking Supervision (Basel Committee on Banking Supervision 2018). Beyond those size indicators, however, it is important to recognize that systemic risk also has a “compositional” nature, due to its dependence on multiple parts that constitute the whole system. This feature has received considerably less attention in the literature, and our work is designed to fill the gap. In particular, we propose a new approach for identifying and understanding the sources of systemic risk in Europe with a focus on the relative contributions of single countries and specific regions.

The novelty of our study consists in adopting the principles of Compositional Data (CoDa) analysis (Aitchison 1982) to investigate the role of European countries as proportions or *parts* of a systemic risk aggregate. A peculiar feature of the compositional approach is that of being specifically designed for research questions which address the distribution of a whole (e.g., shares, allocation) and the relative importance of constituent parts (profile, concentration). Since a relative scale can give better information than an absolute one when it comes to evaluating both large and small proportions (cf. Pawlowsky-Glahn et al. 2015), CoDa methods are particularly effective in distinguishing the threats of potential instability posed by smaller institutions, or peripheral countries, which may not fully emerge from their systemic risk magnitude. This knowledge is ultimately relevant to the design and implementation of regulatory reforms aimed at monitoring risk and allocating capital surcharges commensurate to the systemic weight of various contributors.

The original development of CoDa analysis is related to geosciences and biology problems in which the data are expressed as proportions or concentrations, without an explicit reference to the total size (see Egozcue and Pawlowsky-Glahn 2019, and references therein). Substantial progress has been achieved during the last forty years, and nowadays the term CoDa analysis is used to “stress the fact that what is ultimately compositional is not the data, which may not be parts of a whole or may fail to have a fixed sum, but the research objectives or hypotheses focusing on relative rather than absolute values” (Coenders and Ferrer-Rosell 2020). These features make CoDa analysis a powerful methodology for applications beyond the tradition of hard sciences. Recent studies in management, economics and social disciplines have illustrated in practice the usefulness of compositional methods in addressing a wide variety of problems, ranging from market shares and customer segmentation to tourism and hospitality research, transport systems, risk capital allocation, financial ratios and many more (see, e.g., Linares-Mustarós et al. 2018; Boonen et al. 2019; Grifoll et al. 2019; Coenders and Ferrer-Rosell 2020, and references therein). This framework motivates our interest in a new treatment of systemic risk based on CoDa methods.

Our study relies on systemic risk measures for European financial institutions provided by the Center for Risk Management at the University of Lausanne. The dataset permits the calculation of country-level values of SRISK, a market-based indicator of systemic risk introduced in Acharya et al. (2009, 2012) and recently reviewed by Engle (2018). The properties of SRISK are well-known in the literature, in particular, this measure can be used to identify fragile institutions and countries with a system-wide impact long before a crisis occurs (Stolbov and Shchepeleva 2018) and can be instrumental in forecasting real sector performance (Engle et al. 2014).

Whereas the literature has mainly focused on absolute values of SRISK, in this work we try to push the analysis one step further. Viewing the different European countries as parts of a compositional dataset, we analyze the joint distribution of their SRISK shares using the CoDa methodology and we arrive at a breakdown of systemic risk in Europe into proportional constituents, with interesting links to specific subsets of nations and geographic areas. Our empirical study is developed along the following lines. We first transform the observations into logratio type coordinates and retrieve their location-scale characteristics. Then, the new coordinates are processed with multivariate statistical techniques to discover associations among parts in the SRISK composition and possibly detect underlying patterns. Specifying a hierarchical tree structure (CoDa-dendrogram), we provide a novel interpretation of results in terms of a set of conveniently defined coordinates, or *balances*. Based on a CoDa measure of inequality recently introduced in Egozcue and Pawlowsky-Glahn (2019), we also show how these balance-coordinates contribute to the dynamics of SRISK inequality in Europe during the period 2008–2021.

The outcomes of the empirical analysis show some specific benefits of the proposed CoDa approach in clarifying how systemic risk originates and spreads across different European countries and macro-regions. First, a novel finding of our study is that relatively small parts associated with non-core nations appear to be the main drivers of logratio variability for European SRISK compositions during the period 2008–2021. This clarifies the role of some peripheral countries as sources of potential instability for the whole European economy. Second, although the evolution of SRISK shares is quite complex, a multivariate CoDa analysis permits the identification of a few subcompositions in which the proportion of parts is rather stable over time and is not significantly influenced by other parts in the system. Additionally, the inclusion of a time factor in CoDa-dendrograms highlights a reduction in the total variance of SRISK compositions after the introduction of the European Banking Union and the reform of Basel Accords for the regulation for G-SIFIs. A further contribution of the present work relates to the use of CoDa methods in conjunction with alternative SRISK scenarios. Using a CoDa measure of inequality, we detect some underlying trends in balance-coordinates associated with core and peripheral countries, which reflect possible risk concentration issues inherent in the time dynamics of SRISK. This information is not immediately evident in absolute measures of systemic risk magnitude, as it pertains to the relative balance between subsets of parts in SRISK compositions. Overall, these findings suggest that CoDa methods represent a useful complement to conventional measures, with a view to deepen our understanding of systemic risk sources and reinforce the monitoring process carried out by supervisory authorities.

The paper is organized as follows. Section 2 outlines the principles of CoDa analysis and introduces the methods used throughout the paper. Following a description of the dataset and a brief review of SRISK, Sect. 3 presents the empirical application. In particular, Sect. 3.1 contains an exploratory study focusing on four prominent contributors of systemic risk in the European Union, while Sect. 3.2 extends the compositional analysis to the whole sample of European countries. Then, Sect. 3.3 describes the inequality dynamics of SRISK compositions in Europe and links it to the contributions of peculiar subsets of core and peripheral nations. The results are discussed in Sect. 4, which also provides some concluding remarks.

## Methodology

The analysis performed in this paper largely relies on Compositional Data methodology. Compositional Data (CoDa) are treated as multivariate observations where relative rather than absolute information is relevant, thus they represent a quantitative description of the parts of some whole. In this context, the relevant information is in the proportions among the parts and not in their absolute values or in their sum. The basic elements in compositional techniques are the compositions.

### Definition 1

A composition is a real-valued vector with all strictly positive components. A *D*-part composition is a class of equivalence which contains all the *compositionally equivalent* vectors in ${\mathbb {R}}^D$, where two compositions ${\textbf{x}}=(x_1,x_2,\dots , x_D)$ and ${\textbf{y}}=(y_1,y_2,\dots , y_D)$ are compositionally equivalent if there exists a positive constant $\lambda \in {\mathbb {R}}^+$ such that ${\textbf{x}}=\lambda \cdot {\textbf{y}}$.

A suitable sample space for the equivalence classes is represented by the *D*-part simplex:

1 $$\begin{aligned} {\mathbb {S}}^D=\left\{ (x_1,x_2,\dots ,x_D)\in {\mathbb {R}}^D:\;x_i>0, i=1,2,\dots ,D;\;\sum _{i=1}^D x_i=\kappa \right\} , \end{aligned}$$

where $\kappa$ is a positive arbitrary constant. Refer to Pawlowsky-Glahn et al. (2015) and Egozcue and Pawlowsky-Glahn (2019) and references therein for further details. Usually in compositional analysis, the vectors of proportions (which sum to 1) are used as representatives of an equivalence class. To identify such elements, the following definition of *closure* is considered, choosing $\kappa =1$.

### Definition 2

The closure (to $\kappa$) of the *D*-part composition ${\textbf{x}}=(x_1,x_2,\dots ,x_D)$ is defined by:

$$\begin{aligned} {\mathscr {C}}({\textbf{x}})=\left( \frac{\kappa \cdot x_1}{\sum _{i=1}^Dx_i},\frac{\kappa \cdot x_2}{\sum _{i=1}^Dx_i},\dots ,\frac{\kappa \cdot x_D}{\sum _{i=1}^Dx_i}\right) . \end{aligned}$$

By introducing the two operations of *perturbation* and *powering*, the simplex ${\mathbb {S}}^D$ defined in formula (1) can be proved to be an Euclidean vector space (Pawlowsky-Glahn et al. 2015), with the inner product

$$\begin{aligned} <{\textbf{x}},{\textbf{y}}>_A=\frac{1}{2D}\sum _{i=1}^D \sum _{j=1}^D\left( \ln \frac{x_i}{x_j}\ln \frac{y_i}{y_j}\right) , \end{aligned}$$

the norm

2 $$\begin{aligned} \Vert {\textbf{x}} \Vert _A=\sqrt{\frac{1}{2D}\sum _{i=1}^D\sum _{j=1}^D\left( \ln \frac{x_i}{x_j}\right) ^2}, \end{aligned}$$

and the distance

$$\begin{aligned} d_A({\textbf {x}},{\textbf {y}})=\sqrt{\frac{1}{2D}\sum _{i=1}^D\sum _{j=1}^D \left( \ln \left( \frac{x_i}{x_j}\right) -\ln \left( \frac{y_i}{y_j}\right) \right) ^2}, \end{aligned}$$

where $${\textbf{x}}=(x_1,x_2,\dots ,x_D), {\textbf{y}}=(y_1,y_2,\dots ,y_D) \in {\mathbb {S}}^D$$.

The aforementioned definitions characterize the basic elements to build a particular geometry that in Pawlowsky-Glahn and Egozcue (2001) is called the *Aitchison geometry on the simplex*. Such geometry is valuable for analyzing a compositional dataset.

A typical compositional dataset ${\textbf{X}}$ is a sample of *n* observations of *D*-part compositions ${\textbf{X}}=({\textbf{x}}_1,{\textbf{x}}_2,\dots ,{\textbf{x}}_n)'$, with ${\textbf{x}}_i=(x_{i1},x_{i2},\dots , x_{iD}),\; i=1,2,\dots , n$. In such a dataset, the standard statistical descriptive measures, based on the real Euclidean structure, applied to Compositional Data may lead to erroneous conclusions (Grifoll et al. 2019). To overcome this issue, a set of descriptive measures based on the Aitchison geometry can be used.

### Definition 3

An indicator of central tendency for the dataset ${\textbf{X}}$ is the closed geometric mean. This vector is called *center*, and it can be defined by

$$\begin{aligned} \text{ cen }({\textbf{X}})= {\mathscr {C}}(g_1,g_2,\dots ,g_D), \end{aligned}$$

where $g_j$ is the geometric mean of the *n* observations related to the *j*-th component of the vectors in ${\textbf{X}}$:

$$\begin{aligned} g_j=\left( \prod _{i=1}^{n}x_{ij}\right) ^{1/n},\qquad j=1,2,\dots ,D. \end{aligned}$$

The dispersion in a compositional dataset ${\textbf{X}}$ can be described by the variation matrix, defined by:

$$\begin{aligned} T= \begin{pmatrix} 0 &{}\quad t_{12}&{}\quad \cdots &{}\quad t_{1D} \\ t_{21} &{} 0 &{}\cdots &{}t_{2D} \\ \vdots &{}\vdots &{}\ddots &{}\vdots \\ t_{D1} &{}t_{D2} &{}\cdots &{}0 \end{pmatrix},\quad t_{ij}=var\left( \ln \frac{x_i}{x_j}\right) . \end{aligned}$$

The variation matrix can be summarized in a single measure, the *total variance*.

### Definition 4

The total variance of the compositional sample ${\textbf{X}}$ is a measure of global dispersion of ${\textbf{X}}$, since it is obtained by the variation matrix *T*. It is defined by:

$$\begin{aligned} \text{ totvar }({\textbf{X}})=\frac{1}{2D}\sum _{i=1}^D\sum _{j=1}^D var\left( \ln \frac{x_i}{x_j}\right) =\frac{1}{2D}\sum _{i=1}^D\sum _{j=1}^D t_{ij}. \end{aligned}$$

Since the information provided by compositions is relative, in the CoDa approach the so-called *principle of working in coordinates* has been introduced and developed (Mateu-Figueras et al. 2011; Grifoll et al. 2019). Following such principle, the compositions are transformed into real vectors in order to exploit the usual Euclidean structure. In the literature there are several transformations based on the logratios: the additive logratio (*alr*), the centered logratio (*clr*), the isometric logratio (*ilr*). For a detailed overview on these coordinate transformations, refer to Aitchison (1982), Pawlowsky-Glahn et al. (2015), Egozcue et al. (2003), Mateu-Figueras et al. (2011), Filzmoser et al. (2018), among many others. The *clr* transformation is basically characterized by two fundamental properties: the first one is that it does not change the number of parts, since a *D* composition is transformed into a vector in ${\mathbb {R}}^D$. The second one is that it preserves the distances and the angles, meaning that the Aitchison distance in the simplex of two compositions is equal to the usual Euclidean distance of the corresponding transformed vectors in ${\mathbb {R}}^D$. This feature is very suitable for exploratory analysis based on metrics, like *clr*-biplots. Thus, in this paper we use the centered logratio transformation, defined as follows.

### Definition 5

The centered logratio transformation (*clr*) of a composition ${\textbf{x}}=(x_1,x_2,\dots ,x_D)$ is given by

$$\begin{aligned} clr({\textbf{x}})=\ln \left( \frac{x_1}{g_m({\textbf{x}})},\frac{x_2}{g_m({\textbf{x}})},\dots , \frac{x_D}{g_m({\textbf{x}})}\right) , \end{aligned}$$

where $g_m({\textbf{x}})$ is the geometric mean of the *D* parts, defined by:

$$\begin{aligned} g_m({\textbf{x}})=\left( \prod _{i=1}^{D}x_i\right) ^{1/D}. \end{aligned}$$

In practice, it is often of interest to investigate the behavior of non-overlapping groups of parts within a composition. This can be accomplished through the construction of an orthonormal system of coordinates in ${\mathbb {S}}^D$, called *balances* (a peculiar type of *ilr* coordinates), which provide an interpretation of the groups in terms of relative information (see, e.g., Egozcue and Pawlowsky-Glahn 2019).

### Definition 6

A balancing element in ${\mathbb {S}}^D$ is a *D*-part composition ${\textbf{e}} = (e_1, e_2,\dots , e_D)$ whose *clr* transform ${\textbf{u}} = clr({\textbf{e}})$, satisfies the following properties:

(i) $$\sum _{i=1}^{D} u_i = 0$$;
(ii) $\Vert {\textbf{u}} \Vert _E = \Vert {\textbf{e}} \Vert _A =1$, where $\Vert \cdot \Vert _E$ denotes the usual Euclidean norm;
(iii) the components of ${\textbf{u}}$ only have three possible values: $n_+ \ge 1$ positive values equal to $a_+$, $n_- \ge 1$ negative values equal to $a_-$ and $D - n_+ - n_- \ge 0$ zero values.

The length of the orthogonal projection of a composition ${\textbf{x}} \in {\mathbb {S}}^D$ onto the balancing element ${\textbf{e}}$ is the balance $b({\textbf{x}})= <{\textbf{x}},{\textbf{e}}>_A$. Equivalently, $b({\textbf{x}})$ is a balance if it can be written as

$$\begin{aligned} b({\textbf{x}}) = w \ln \frac{g_m({\textbf{x}}_+)}{g_m({\textbf{x}}_-)}, \quad w = \sqrt{\frac{n_+ n_-}{n_+ + n_-}}, \end{aligned}$$

where ${\textbf{x}}_+$, ${\textbf{x}}_-$ denote two sets of parts in ${\textbf{x}}$ consisting, respectively, of $n_+$ and $n_-$ elements, with $n_+ + n_- \le D$.

An orthonormal basis of ${\mathbb {S}}^D$ made of balancing elements can be constructed using the Sequential Binary Partition (SBP) procedure, clearly described in Pawlowsky-Glahn and Egozcue (2011). The balance-coordinates of a composition ${\textbf{x}}$ corresponding to such a basis are assigned by the isometric logratio (*ilr*) transformation:

$$\begin{aligned} ilr({\textbf{x}}) = V' clr({\textbf{x}}), \end{aligned}$$

with inverse:

$$\begin{aligned} {\textbf{x}} = {\mathscr {C}} \left( \exp (V \, ilr({\textbf{x}})) \right) , \end{aligned}$$

where *V* is a *D* by $(D-1)$ matrix having the *clr* values of $D-1$ orthonormal balancing elements as columns and $clr({\textbf{x}})$, $ilr({\textbf{x}})$ are considered as column vectors with *D* and $D-1$ components, respectively (further mathematical details and examples can be found, e.g., in Filzmoser et al. 2018; Egozcue and Pawlowsky-Glahn 2019 and in the references therein).

In any exploratory CoDa analysis, graphics play an effective role. The most widely used explanatory tools are two: the biplots and the ternary diagrams (also called De Finetti diagrams). The biplots have been introduced in Gabriel (1971) for visualizing the rows and the columns of many different kinds of data matrix, by a rank-2 approximation. In Aitchison and Greenacre (2002), they have been specifically adapted for compositional data, becoming very powerful exploratory tools, largely employed in applications. The biplots are based on the Singular Value Decomposition (SVD) of the centered (or standardized) data matrix. The procedure relies on well-established techniques of Principal Component Analysis (PCA), which were originally formalized for compositional datasets in Aitchison (1983).^1^ To enhance the data analysis, Aitchison and Greenacre (2002) proposed two kinds of biplots: the *form biplot*, which favours the display of the units, and the *covariance biplot*, which favours the display of the variables (Greenacre and Underhill 1982). More details can be found in Pawlowsky-Glahn et al. (2015), Greenacre (2018) and references therein.

A ternary diagram is an equilateral triangle where the compositions can be represented by points. Each vertex corresponds to a part. The composition ${\textbf{x}}=(x_1,x_2,x_3)$ is plotted at a distance $x_1$ from the opposite side of the vertex corresponding to the first part $X_1$, at a distance $x_2$ from the opposite side of the vertex corresponding to the second part $X_2$, and finally at a distance $x_3$ from the opposite side of the last vertex. This means that the closer a point (composition) is located near the border, the closer are the values of one or two parts to zero (Filzmoser et al. 2018).

It is worth recalling that ternary diagrams do not reflect the relationships between compositions in the Aitchison geometry, but in the standard Euclidean geometry, and thus they can be misinterpreted, particularly when the compositions are close to the boundaries of the diagram. To overcome this issue, in some cases, the *centered ternary diagrams* can be useful. A centered ternary diagram is a ternary diagram, obtained after a perturbation of the original compositions by the inverse of their center. The effect of a such operation is an optimized rescaling, which leads to the centering of the dataset around the baricenter of the ternary diagram (von Eynatten et al. 2002).

The ternary (and also quaternary) diagrams are very useful tools to find relationships among the observations and to identify compositional patterns in the data. For a detailed description of De Finetti diagrams and their role in compositional analysis, we refer the reader to Greenacre (2018), Pawlowsky-Glahn et al. (2015), and Filzmoser et al. (2018).

A concept that is closely related to the relative importance of parts in a composition is that of inequality, or concentration. Recently, Egozcue and Pawlowsky-Glahn (2019) have proved that the square of the Aitchison norm defined in formula (2) over the number of parts in a compositional vector satisfies a list of requirements similar to those defining Atkinson’s inequality indexes (cf. Shorrocks 1980). In particular, the measure has large values when the shares tend to concentrate in a few units, and small values when shares spread over all units with similar proportions; it is symmetric in the arguments, scale invariant and consistent with the Pigou-Dalton principle of transfers. For a *D*-part composition ${\textbf{x}}$, Egozcue and Pawlowsky-Glahn (2019) have formulated this new inequality index as

3 $$\begin{aligned} A_I^2({\textbf{x}}) = \frac{1}{D} \Vert {\textbf{x}} \Vert _A ^2 = \frac{1}{D} \sum _{i=1}^D (clr_i({\textbf{x}}))^2 = \frac{1}{D} \sum _{i=1}^{D-1} (ilr_i({\textbf{x}}))^2, \end{aligned}$$

showing two different ways of decomposing the square norm of a composition in terms of either its *clr*- or its *ilr*-coordinates. The latter can be associated with an orthonormal basis of ${\mathbb {S}}^D$, thus allowing for an interpretation of inequality contributions in terms of square balances (cf. Egozcue and Pawlowsky-Glahn 2019, for a detailed description and further properties of $A_I^2$).

## Data analysis

We conduct an empirical study based on country-level measures of systemic risk provided by the Center for Risk Management at the University of Lausanne (CRML), Switzerland (http://www.crml.ch). Our work belongs to the recent research stream that uses public statistical data to enhance the understanding and the monitoring of systemic risk (cf. Stolbov and Shchepeleva 2018). The computations involving compositional techniques are performed with the freeware CoDaPack (Comas-Cufí and Thió-Henestrosa 2011), available at the website http://www.compositionaldata.com/.

The reference indicator of systemic risk computed by CRML is the SRISK measure introduced in Acharya et al. (2009, 2012) and subsequently extended in a number of papers (cf. Engle 2018, for a detailed review). At a given moment *t*, the SRISK of a financial institution *i* is defined as:

$$\begin{aligned} \text {SRISK}_{it} = max \lbrace 0; \underbrace{ k \left[ D_{it} + (1- \text {LRMES}_{it}) W_{it} \right] }_{\textit{Required Capital}} - \underbrace{ (1- \text {LRMES}_{it}) W_{it}}_{\textit{Available Capital}}\rbrace , \end{aligned}$$

where *k* is the prudential capital ratio established by regulatory authorities, $D_{it}$ is the book value of total liabilities, $W_{it}$ denotes market capitalization and LRMES is the Long-Run Marginal Expected Shortfall, which corresponds to the expected drop in equity value conditional on the market falling by more than $40\%$ within the next six months (see, e.g., Acharya et al. 2012; Brownlees and Engle 2017, for a detailed description of the methodology).

Nation-wide measures of SRISK are computed by aggregation of metrics available for individual firms. As argued in Engle et al. (2014), these aggregate measures provide an early warning signal of distress in the real economy and a reasonable prediction for the cost of a bailout. Furthermore, the ratio of the SRISK pertaining to a specific country to the overall SRISK of the financial system represents the systemic risk share (*part*) associated with that country, an information that can be properly processed by a CoDa approach.

Our study focuses on a sample of ten European economies (Belgium, Denmark, France, Germany, Greece, Italy, Netherlands, Spain, Switzerland, UK) with yearly SRISK observations recorded at end-December for the period 2002–2021. All data are expressed in billion Euros. In analogy with most applications of CoDa methods, the sample does not cover the whole European continent and thus the ten parts forming our SRISK compositions are only a subset of those possible. Nonetheless, CoDa analysis relies on a fundamental principle of *subcompositional coherence*, which guarantees that a compositional study performed on a subset of parts is always coherent with the same analysis applied to the whole composition. The full set of conditions underlying this principle can be found in the classical literature on compositional data.

**Fig. 1:**
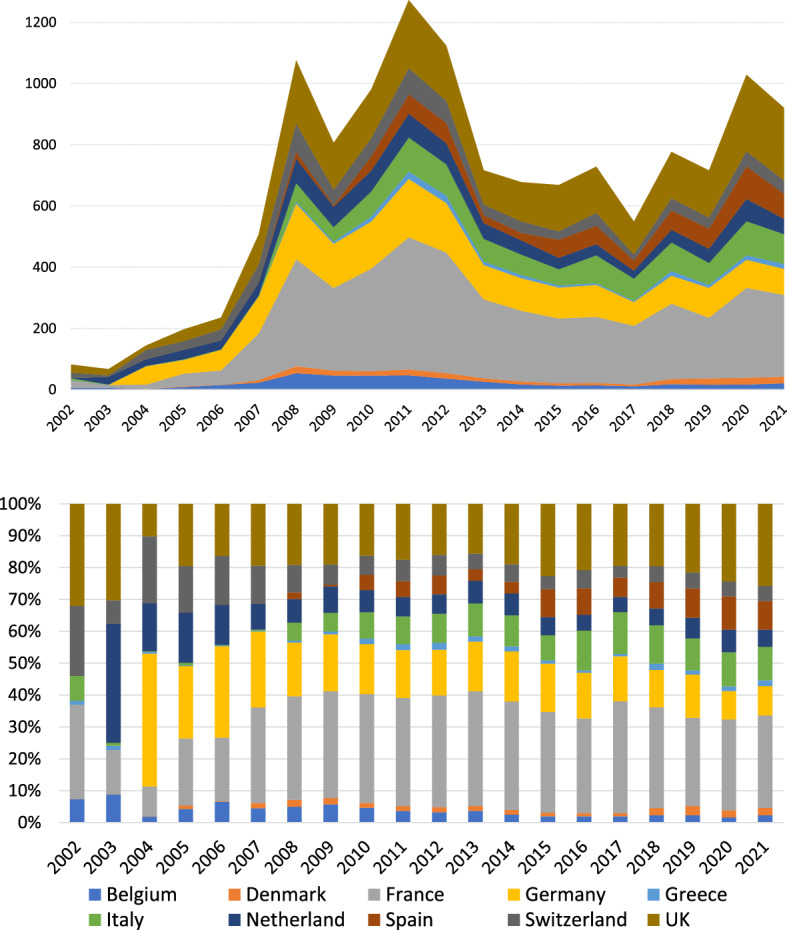
Evolution of SRISK in the 10 European countries: total amounts, in billion Euros (top panel); percentage compositions (bottom panel)

Figure 1 depicts the evolution of SRISK over the sample period both in absolute values (top panel) and in percentage compositions (bottom panel). What clearly emerges from the compositional perspective is that systemic risk was concentrated on a very small number of parts in the early 2000s, but effectively became a global issue when the consequences of the GFC reached European institutions in 2008. For this reason, and for critical issues arising with irregular data,^2^ our CoDa analysis of systemic risk in Europe will be focused on the period 2008–2021. Based on features of the dataset and in accordance with recent literature on the topic (cf. Black et al. 2016), we further distinguish two different subperiods: (a) years 2008–2012, corresponding to the spread of the GFC to Europe and the consequent outbreak of the sovereign debt crisis, (b) years 2013–2021, characterized by the introduction of the European Banking Union and the preparatory phase of the new regulation for G-SIFIs promoted by the Basel Committee on Banking Supervision (BCBS).

### Subcompositional analysis of SRISK in major EU countries

As mentioned in the Introduction, the literature on systemic risk in European financial institutions has identified four major contributors within the Euro area: France, Germany, Italy and Spain (Petrella et al. 2018). Among them, France has historically occupied a prominent position in terms of systemic risk magnitude, followed by Germany. During the sovereign debt crisis, however, a notable increase in systemic importance came from the Italian and Spanish banks, in spite of their comparatively smaller sizes. This evidence suggests that “concerns regarding relatively smaller banks in these southern European countries can still have significant systemic risk implications for the rest of Europe, possibly due to the high correlation or contagion effect” (Black et al. 2016). Based on the above considerations, we begin our empirical study with a focus on the subcomposition formed by France, Germany, Italy and Spain. Following Pawlowsky-Glahn et al. (2015), the adoption of a compositional perspective is worthwhile in this framework since a relative scale can give better information than an absolute one when it comes to evaluating the comparative dynamics of smaller and larger systemic risk proportions.

**Fig. 2:**
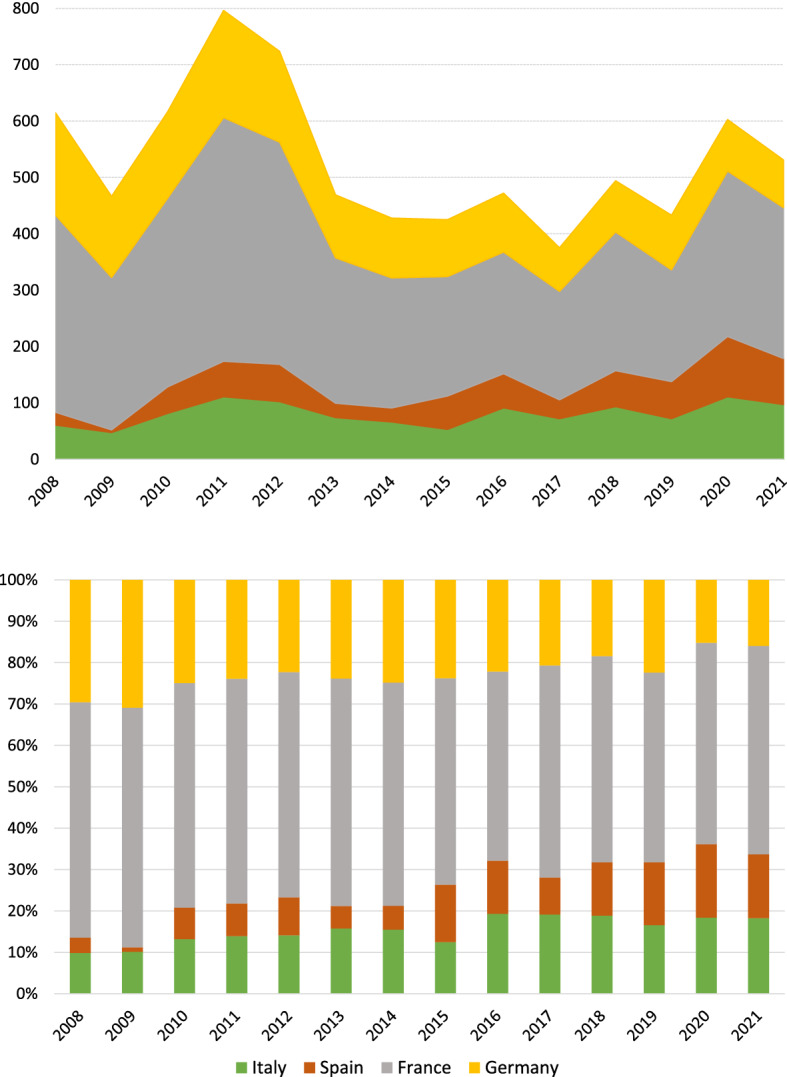
Evolution of SRISK in the four-part subcomposition formed by France, Germany, Italy and Spain: total amounts, in billion Euros (top panel); percentage compositions (bottom panel)

As shown in Fig. 2 (top panel), the raw evolution of SRISK in these countries has a sharp increase between 2009 and 2011, followed by a rapid reduction after 2012. Then, SRISK stabilizes in Germany around a value of 100 billion Euros whereas France alternates a sequence of peaks and troughs in the SRISK dynamics. Italy and Spain experience a progressive increase in SRISK after 2015 and eventually exceed Germany in 2020, corresponding to the outbreak of Covid-19 pandemics in Europe. Indeed, the bar chart of relative contributions (Fig. 2, bottom panel) confirms that the aggregate weight of Italy and Spain (Euro-Mediterranean countries) has more than doubled in the observed period, raising from $14\%$ in 2008 to $33\%$ in 2021 with an increasing presence of Spain. Focusing on the Euro-Continental region, the importance of Germany relative to France has remained approximately stable over time.

Tables 1 and 2 contain, respectively, the sample center and the variation matrix of the four-part subcomposition of SRISK. The center highlights a predominance of France, with a geometric mean exceeding a $53\%$ SRISK share over the sample period. The maximum variability of the composition is associated with Spain, possibly as a consequence of SRISK fluctuations caused by the sovereign debt crisis and subsequent instability. The smallest logratio variance is observed between France and Germany, suggesting a proportional behavior of these parts in the observed SRISK composition.

**Table 1:** The center of the four-part subcomposition

Country	Center
France	0.5324
Germany	0.2295
Italy	0.1545
Spain	0.0836

**Table 2:** Logratio variances of the four-part subcomposition formed by France (F), Germany (D), Italy (I) and Spain (E)

	F	D	I	E	Clr-variance
F	0	0.0256	0.0749	0.6091	0.0536
D	0.0256	0	0.1530	0.7784	0.1155
I	0.0749	0.1530	0	0.3393	0.0180
E	0.6091	0.7784	0.3393	0	0.3079
Total variance					0.4950

**Fig. 3:**
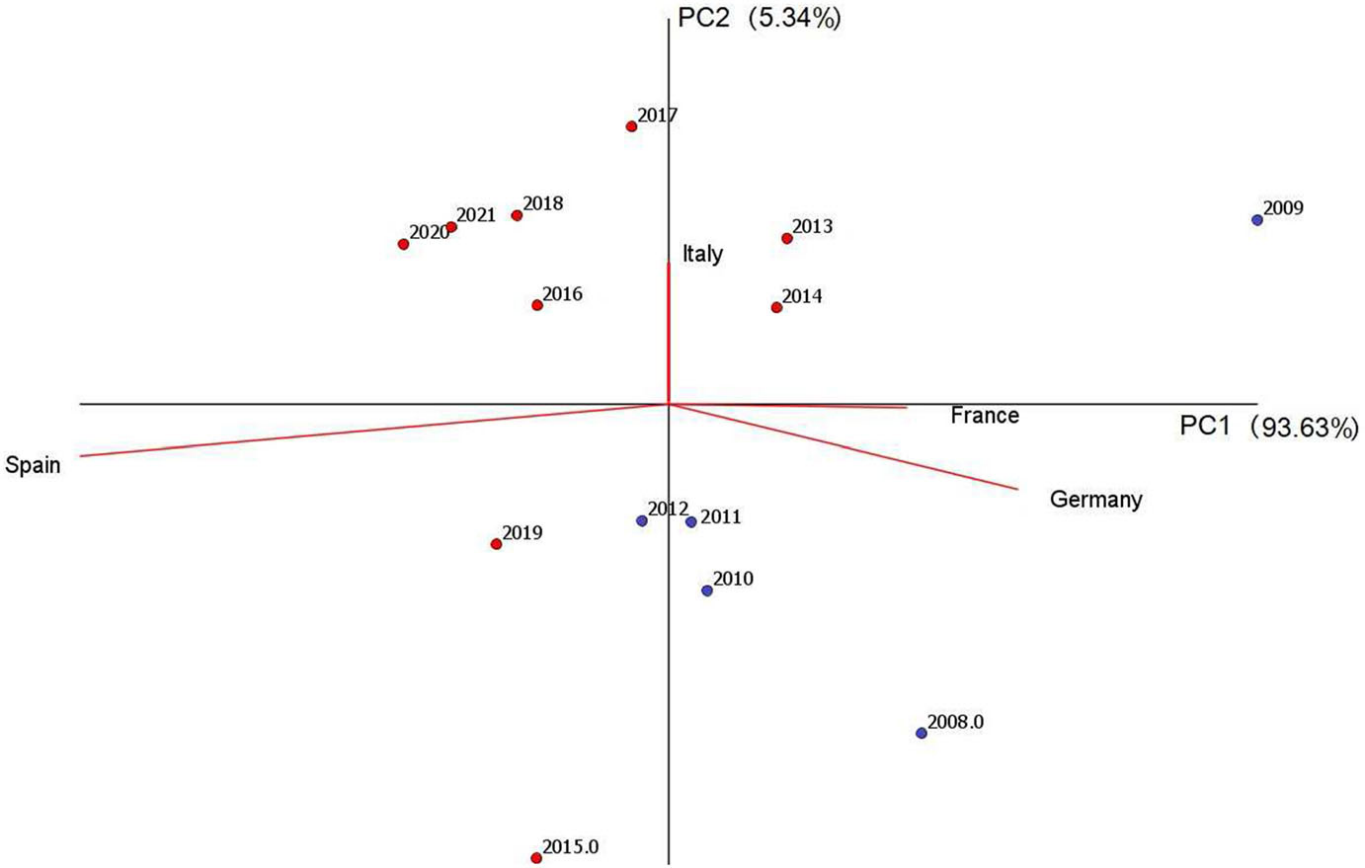
Covariance biplot of the SRISK composition France–Germany–Italy–Spain. The projection accounts for $98.97\%$ of total variance, and the proportions of variance captured by the first and second principal component are shown in brackets. Blue dots refer to the period 2008–2012 (GFC and sovereign debt crisis), red dots correspond to the period 2013–2021 (new regulation for G-SIFIs) (color figure online)

The exploratory analysis of the dataset can be further enhanced by a Compositional Principal Component Analysis (CoDa-PCA), which can be broadly described as a conventional PCA performed on centered logratios (see, e.g., Coenders and Ferrer-Rosell 2020, and references therein). The corresponding covariance biplot (Aitchison and Greenacre 2002) is displayed in Fig. 3. The purpose of this biplot is to simultaneously represent individual compositions as data points (with year labels) and *clr*-variables as rays emanating from a common origin (the center of the dataset), after a projection in two dimensions. The visual interpretation permits a deeper understanding of variability and relationships between parts in the compositions. Up to the bidimensional projection, the length of each ray is approximately proportional to the standard deviation of the corresponding *clr*-variable, whereas the distance between the vertices of two rays (*link*) is approximately proportional to the square root of the logratio variance between the corresponding parts. Parts, behaving proportionally in the sample, appear as rays with end-points that are close together. More generally, the cosine of the angle formed by two links is an approximation of the linear correlation coefficient between the corresponding logratio variables (Pawlowsky-Glahn et al. 2015).

In the biplot displayed in Fig. 3, the projection on the first two principal components accounts for about $99\%$ of the total variance, with the first axis representing more than $93\%$. France and Germany have the shortest link, which confirms the smallest variability of the respective logratio (cf. Table 2). The vertices of France, Germany and Italy are nearly collinear, suggesting that the associated subcomposition has a one-dimensional pattern of variability (Pawlowsky-Glahn et al. 2015, p. 74). The behavior of Spain is somehow different. In fact, the position of the Spain vertex and its distance from the origin imply that longer links are needed to connect Spain with all other vertices. This is a consequence of the fact that logratios involving Spain have the highest sample variability (see also Table 2).

**Fig. 4:**
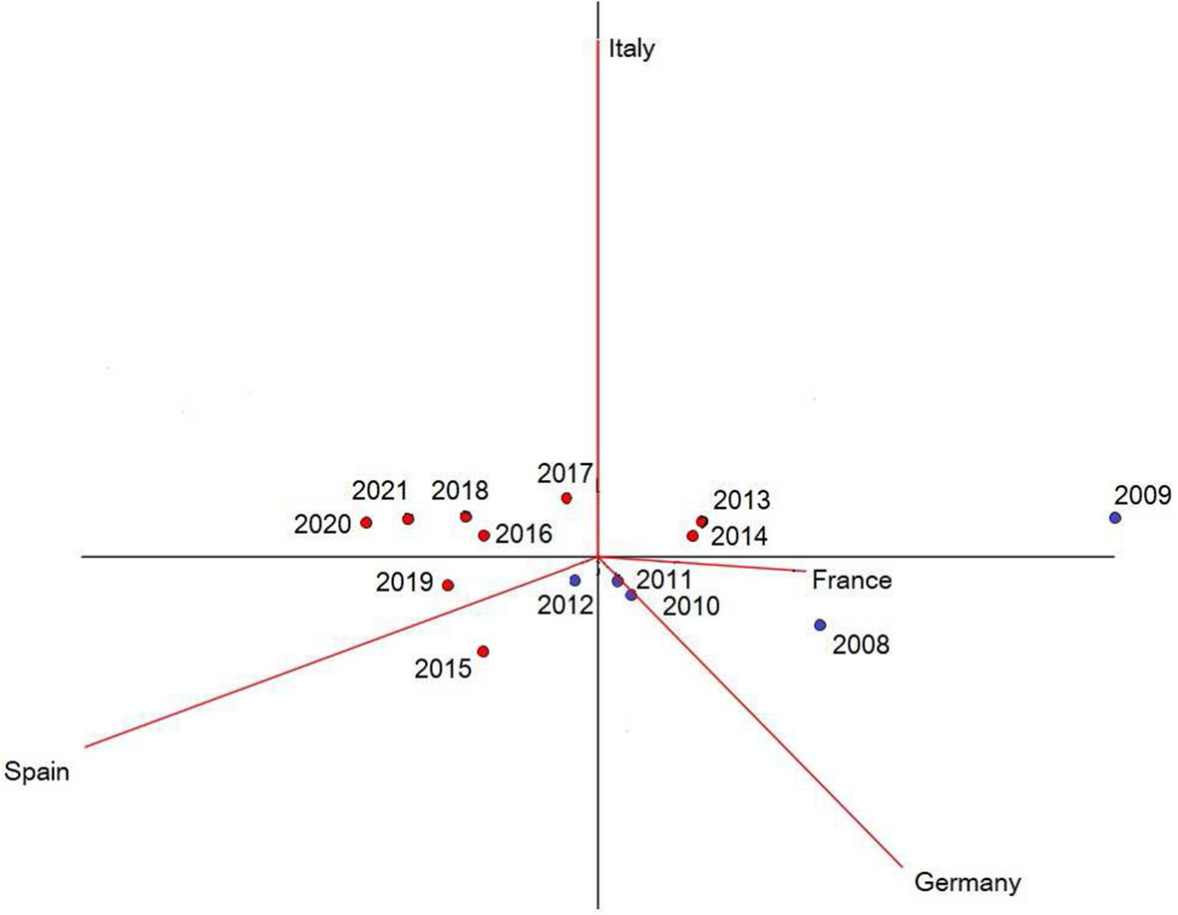
Form biplot of the SRISK composition France–Germany–Italy–Spain. This representation favors the display of years (samples). Blue dots are used for period 2008–2012, red dots for 2013–2021

Applying suitable scale changes along the horizontal and vertical axes of the display, we obtain the form biplot in Fig. 4, which preserves the same quality (i.e. percentage of total variance retained) as the previous covariance biplot but favors the representation of samples, corresponding to years in our dataset (see, e.g., Aitchison and Greenacre 2002 for details). Based on the position of markers in Fig. 4, we observe that the SRISK compositions for 2008 and, more importantly, for 2009, are located quite far from other years, supporting the idea that the outbreak of the GFC in Europe and the consequent sovereign debt crisis had a major impact on the distribution of SRISK shares in the Euro area. Successive years are closer to the center of the biplot, and thus more similar to the center of the compositional sample. A possible way of extending this description would require studying the dataset as a compositional time series, something which is beyond the scope of the present work and is left for future research.

In order to deepen the interpretation of variability and cross-country relationships, an orthonormal basis linked to a Sequential Binary Partition (SBP) is selected. The proposed SBP is encoded through the sign matrix reported in Table 3, which defines a new system of coordinates in ${\mathbb {R}}^{D-1}={\mathbb {R}}^3$ derived from an isometric logratio (*ilr*) transformation of the original components (Pawlowsky-Glahn and Egozcue 2011). Based on the distinction of “core” and “peripheral” countries discussed in the literature on systemic risk in Europe (cf. Black et al. 2016; Petrella et al. 2018, among many others), we consider the following *ilr*-coordinates (*balances*):

$$\begin{aligned} b_1&= \ln \left( \frac{X_{F} X_{D}}{X_{I} X_{E}} \right) ^{1/2},\\ b_2&= \sqrt{\frac{1}{2}} \ln \left( \frac{X_{F}}{X_{D}} \right) ,\\ b_3&= \sqrt{\frac{1}{2}} \ln \left( \frac{X_{I}}{X_{E}} \right) , \end{aligned}$$

where $X_F,X_D, X_I,X_E$ denote the parts associated with France, Germany, Italy and Spain, respectively. The first balance ($b_1$) expresses the comparison between Continental and Euro-Mediterranean countries in terms of the normalized logratio between the geometric mean of France and Germany and that of Italy and Spain. The second and third balances are obtained as normalized logratios between pairs of countries that belong either to Continental Europe ($b_2$) or to the Euro-Mediterranean region ($b_3$). Hence, these balances complete the SBP by differentiating between the contributions of single parts inside each macro-area specified in $b_1$.

**Fig. 5:**
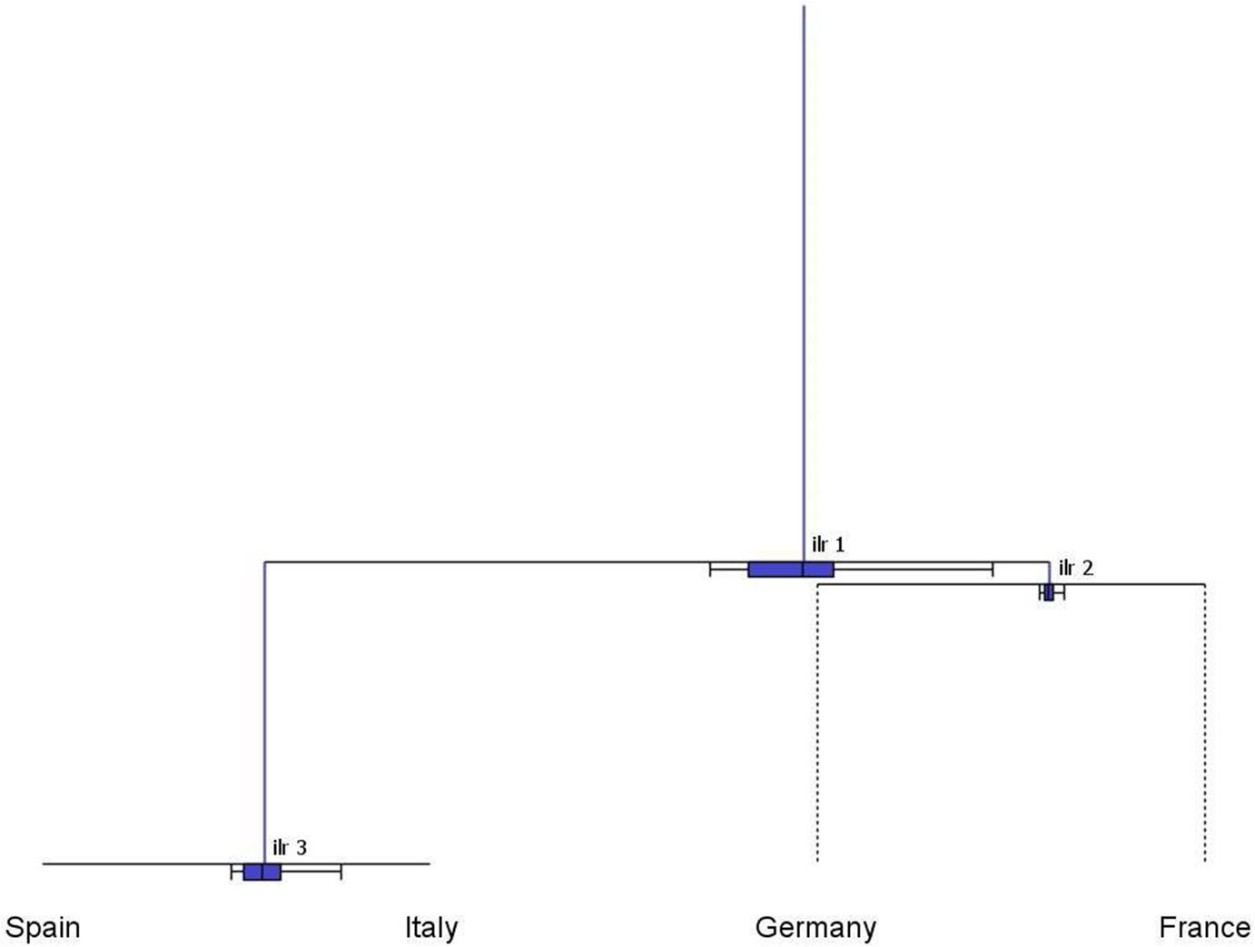
CoDa-dendrogram: balances of the SRISK four-part subcomposition, corresponding to the SBP in Table 3 (years 2008–2021)

The CoDa-dendrogram (Fig. 5) provides a graphical representation of the main characteristics (location and dispersion) for each balance in the SBP, as well as a visualization of the *ilr*-decomposition of the total variance in the form of a tree structure. In a CoDa-dendrogram, vertical segments are proportional to the variance of the respective balance and their lengths add to the total variance of the sample. The intersections between vertical and horizontal segments identify the mean balances, i.e. the coordinates corresponding to the sample center (see Pawlowsky-Glahn and Egozcue 2011 for details).

**Table 3:** Sequential Binary Partition (SBP) for the subcomposition of four major SRISK contributors in the European Union (France–Germany–Italy–Spain)

Balance	France	Germany	Italy	Spain
$b_1$	1	1	$-$ 1	$-$ 1
$b_2$	1	$-$ 1	0	0
$b_3$	0	0	1	$-$ 1

In Fig. 5, the CoDa-dendrogram for the whole sample (2008–2021) allocates the largest portion of total variance to the balance between Continental and Euro-Mediterranean countries, which is associated with the longest vertical segment (*ilr*-1). The third balance (*ilr*-3) also exhibits a large variability, and consequently points to a relevant contribution of the Italy–Spain logratio to the total variance. Conversely, the vertical segment associated to *ilr*-2 is so small that it suggests that the France–Germany logratio is approximately constant across the sample (Egozcue and Pawlowsky-Glahn 2019). Horizontal segments and boxplots in Fig. 5 can be useful to visualize the empirical distribution of balances. In particular, the mean balance between Continental and Euro-Mediterranean countries (*ilr*-1) is shifted towards the former, indicating that the geometric mean of France–Germany is greater than that of Italy–Spain. The associated boxplot is located entirely to the right of the middle point of the horizontal segment, confirming the prevailing role of the Continental region in the first balance. In the second and third balances, the center is respectively shifted towards France (relative to Germany) and Italy (relative to Spain). Looking at boxplots, however, we observe that the comparison between Italy and Spain (*ilr*-3) exhibits greater asymmetry and dispersion relative to the France–Germany balance (*ilr*-2).

**Fig. 6:**
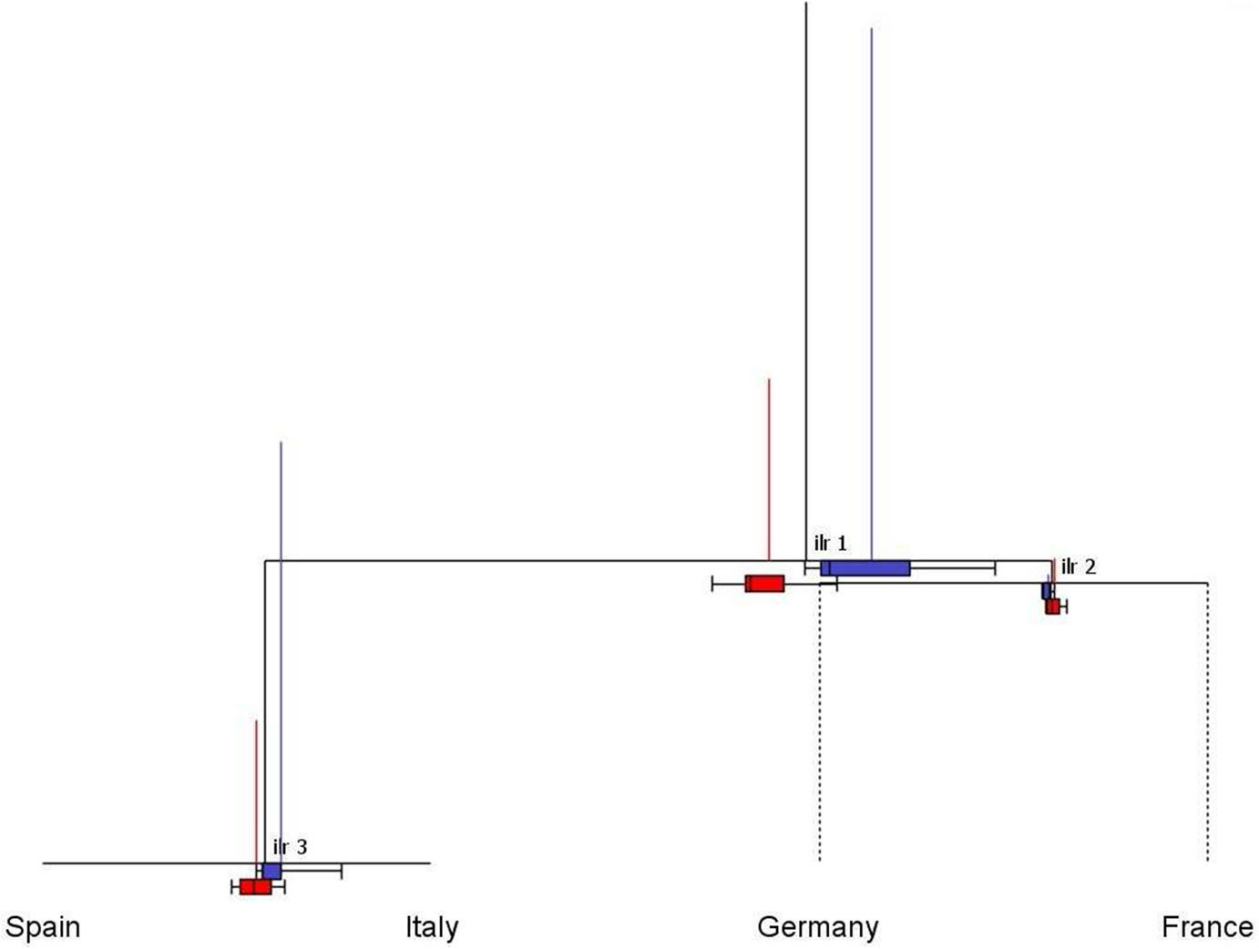
CoDa-dendrogram: balances of the SRISK four-part subcomposition, corresponding to the SBP in Table 3 for the two periods. First period: 2008–2012 (in blue); Second period: 2013–2021 (in red) (color figure online)

Further insights can be gained from the CoDa-dendrogram with the addition of a time evolution factor. Figure 6 explores this possibility classifying the years 2008–2012 as a first period, corresponding to the spread of the Global Financial Crisis (GFC) to Europe and the consequent sovereign debt crisis, and the years 2013–2021 as a second period, characterized by a gradual introduction of the European Banking Union and new regulatory requirements for G-SIFIs. The CoDa-dendrograms pertaining to each period are superposed in Fig. 6 to highlight temporal changes in the empirical distribution of balances. Comparing the blue and red vertical segments, we observe a considerable reduction in the variance contributions of the first and third *ilr*-coordinates, which implies a global decrease in the total variance of the SRISK composition. The first mean balance shows an evolution towards the Euro-Mediterranean region, with lower dispersion and skewness in the associated boxplot. The distribution of the third balance has also become more symmetric, with a left-shift in the mean towards Spain. Interestingly, the second balance reveals a different evolution, with a moderate increase in dispersion and skewness associated with the France–Germany comparison.

### Compositional analysis of SRISK in European financial institutions

After considering the four-part subcomposition formed by France, Germany, Italy and Spain, we now apply CoDa procedures to analyze the full sample of ten European countries for the period 2008–2021. The basic descriptive statistics of the data are presented in Tables 4 and 5, which report, respectively, the center of the ten-part SRISK composition and the variation matrix of pairwise logratios.

**Table 4:** The center of the ten-part composition formed by European countries

Country	Center
Belgium	0.0288
Denmark	0.0173
France	0.3303
Germany	0.1424
Greece	0.0134
Italy	0.0959
Netherlands	0.0641
Spain	0.0519
Switzerland	0.0556
UK	0.2003

**Table 5:** Logratio variances of the ten-part composition formed by European countries, labeled with the following codes: Belgium (B), Denmark (DK), France (F), Germany (D), Greece (GR), Italy (I), Netherlands (NL), Spain (E), Switzerland (CH), United Kingdom (UK)

	B	DK	F	D	GR	I	NL	E	CH	UK	*Clr*-variance
B	–	0.2095	0.1236	0.0885	0.3180	0.3591	0.0948	1.1668	0.0754	0.2476	0.1607
DK	0.2095	–	0.1225	0.1778	0.1776	0.1832	0.0748	0.6587	0.1000	0.0875	0.0715
F	0.1236	0.1225	–	0.0256	0.1594	0.0749	0.0270	0.6091	0.0465	0.0477	0.0160
D	0.0885	0.1778	0.0256	–	0.2569	0.1530	0.0429	0.7784	0.0495	0.1004	0.0597
GR	0.3180	0.1776	0.1594	0.2569	–	0.1677	0.1767	0.5161	0.2060	0.2065	0.1109
I	0.3591	0.1832	0.0749	0.1530	0.1677	–	0.1394	0.3393	0.1736	0.0668	0.0581
NL	0.0948	0.0748	0.0270	0.0429	0.1767	0.1394	–	0.7229	0.0339	0.0599	0.0296
E	1.1668	0.6587	0.6091	0.7784	0.5161	0.3393	0.7229	–	0.7507	0.4741	0.4940
CH	0.0754	0.1000	0.0465	0.0495	0.2060	0.1736	0.0339	0.7507	–	0.0920	0.0452
UK	0.2476	0.0875	0.0477	0.1004	0.2065	0.0668	0.0599	0.4741	0.0920	–	0.0306
Total variance											1.0762

These statistics confirm the prominent role of France, with a $33.03\%$ average SRISK share, followed by the UK ($20.03\%$), Germany ($14.24\%$) and Italy ($9.59\%$). Spain is a smaller contributor of SRISK on average ($5.19\%$), but has the largest logratio variances with all other countries. The lowest entries in the variation matrix are associated with Switzerland and Netherlands, whereas the UK has an alternating behavior (smaller logratio variances associated with Belgium and Greece, larger logratio variances with France, Netherlands, Italy and Denmark).

**Fig. 7:**
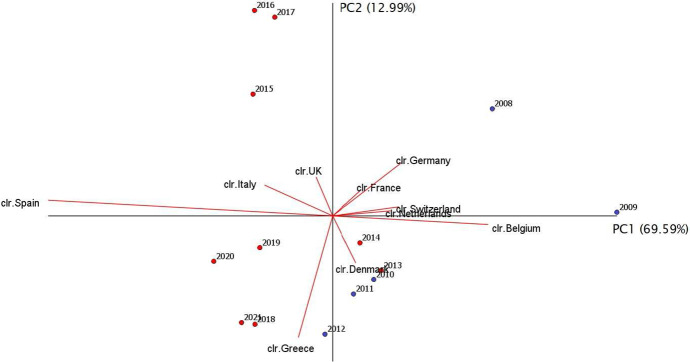
Covariance biplot of the SRISK composition for the 10 European countries. The projection accounts for $82.58\%$ of total variance, and the proportions of variance captured by the first and second principal component are shown in brackets. Blue dots refer to the period 2008–2012, red dots to the period 2013–2021 (color figure online)

A CoDa-PCA is performed on the *clr*-transformed SRISK compositions, yielding the covariance biplot displayed in Fig. 7. This plot represents a bi-dimensional projection on the plane formed by the first two principal components, which globally account for $82.58\%$ of the total variance.^3^ In the SRISK compositions, the first principal component differentiates the Continental Region (mainly Belgium, Netherlands, Switzerland, Germany) from Euro-Mediterranean countries (Italy and Spain), while the second principal component essentially distinguishes Greece and Denmark (minor contributors to SRISK) from France, Germany, Italy and UK (major contributors). The longest ray is associated with Spain, corresponding to the highest variability of centered logratios. Although the interpretation of rays as indicators of the variance of one single part should be generally avoided (Pawlowsky-Glahn et al. 2015), in this case we observe that Spain has long links with all parts in the compositions (in particular, with countries in the Continental Region). This implies that logratios involving Spain have the largest contributions to total variance, as already emerged from Table 5. Another relevant distance is between Greece and Germany, which considerably lays along the second principal component.

More important than the position of single rays, the complete set of links reflects the compositional covariance structure and provides useful insights into subcompositional variability and possible independence between parts. Focusing on countries in the Continental Region, the proximity of France and Germany properly reflects their moderate logratio variability (Table 5). A similar situation occurs with Netherlands and Switzerland, which display the shortest link corresponding to one of the smallest logratio variances in the SRISK composition.

Considering the cosine of angles between pairs of links permits an approximate evaluation of the linear correlation between the corresponding logratios. Interestingly, the UK–Denmark link is nearly orthogonal to the France–Germany link, suggesting that the corresponding logratios should be checked for zero correlation. Direct computation indeed confirms that the linear correlation between the logratios of UK–Denmark and France–Germany equals $-\,0.027$. Furthermore, the Netherlands–Switzerland link appears approximately orthogonal to a number of links in the biplot, suggesting again a low correlation between the corresponding logratios (Table 5). Indeed, the logratio Netherlands–Switzerland has a linear correlation coefficient equal to 0.0327 with the logratio Italy–Greece, $-\,0.0056$ with the logratio Spain–Greece, $-\,0.1184$ with the logratio Belgium–Spain, and 0.0624 with the logratio UK–Denmark.

**Fig. 8:**
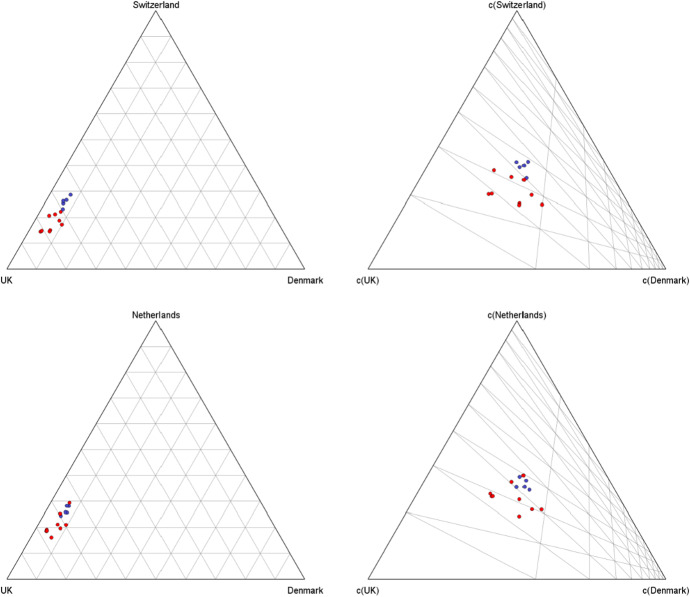
Ternary diagrams (left panels) and centered ternary diagrams (right panels) of the subcompositions Switzerland–UK–Denmark and Netherlands–UK–Denmark. Blue dots: first period (2008–2021), red dots: second period (2013–2021) (color figure online)

As aforementioned, ternary diagrams can also be used to visualize the displacement of some subcompositions. In the left panels of Fig. 8 the ternary diagrams of the subcompositions Switzerland–UK–Denmark and Netherlands–UK–Denmark are drawn. In both cases, the data lie quite close to the boundary of the simplex and are quite compressed. Thus, to better visualize the internal structure of the data, the centered ternary diagrams are shown in the right panels. It is interesting to remark that the data of the two subcompositions share very similar locations in the diagrams.

In order to investigate specific differences between subgroups of countries, we introduce a new system of orthonormal coordinates in ${\mathbb {R}}^{D-1} = {\mathbb {R}}^{9}$ linked to a Sequential Binary Partition. The proposed SBP is encoded through the sign matrix in Table 6, which determines a hierarchical partition of nations starting from an isometric logratio transformation of their parts in the SRISK composition. The corresponding balances were inspired by the following criteria:

- balance $b_1$ compares the geometric mean of France–Germany–Italy–Spain to that of the remaining countries. The key role of the first subcomposition within the European Union suggests a moderate dependence with other parts, and consequently a high contribution of this balance to the total variance (cf.  Grifoll et al. 2019 for details);
- balances $b_2$-$b_4$ replicate the SBP partition used in Sect. 3.1 for the France–Germany–Italy–Spain subcomposition;
- balance $b_5$ differentiates the remaining countries in two groups according the reciprocal positions and distances of their veritices in the covariance biplot (Fig. 7). This is done by considering the normalized logratio between the geometric mean of Belgium–Netherlands–Switzerland (which are nearly aligned along the direction of the first principal axis) and that of Denmark–Greece–UK (which are somehow closer to the second principal axis but possibly oriented in different directions);
- balances $b_6$-$b_9$ complete the SBP by further differentiating between subgroups or single parts involved in the previous step.

**Table 6:** Sequential Binary Partition (SBP) for the SRISK composition with ten European countries

Balance	B	DK	F	D	GR	I	NL	E	CH	UK
$b_1$	$-$ 1	$-$ 1	1	1	$-$ 1	1	$-$ 1	1	$-$ 1	$-$ 1
$b_2$	0	0	1	1	0	$-$ 1	0	$-$ 1	0	0
$b_3$	0	0	1	$-$ 1	0	0	0	0	0	0
$b_4$	0	0	0	0	0	1	0	$-$ 1	0	0
$b_5$	$-$ 1	1	0	0	1	0	$-$ 1	0	$-$ 1	1
$b_6$	0	1	0	0	$-$ 1	0	0	0	0	1
$b_7$	0	$-$ 1	0	0	0	0	0	0	0	1
$b_8$	$-$ 1	0	0	0	0	0	1	0	1	0
$b_9$	0	0	0	0	0	0	1	0	$-$ 1	0

**Fig. 9:**
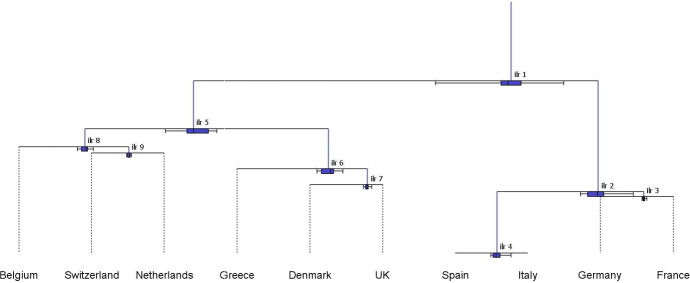
CoDa-dendrogram: balances of the SRISK composition with ten European countries according to the SBP in Table 6 (whole sample)

The CoDa-dendrogram in Fig. 9 illustrates the outcome of the previous SBP, visualizing in a hierarchical tree structure the *ilr*-decomposition of total variance. The length of vertical bars, which is proportional to the variance of the corresponding balances, shows considerable differences across the various coordinates. In particular, balances $b_1$, $b_2$ and $b_4$ capture the highest fractions of total variance, confirming the prominent contribution of France–Germany–Italy–Spain to the overall variability of SRISK compositions. Indeed, due to subcompositional coherence, the tree structure of balances $b_2$-$b_4$ replicates exactly the CoDa-dendrogram displayed in Fig. 5. For the *ilr*-1 coordinate, the intersection between the vertical and horizontal bars indicates that the mean balance is clearly shifted right, reflecting the prevailing influence of France–Germany–Italy–Spain on SRISK compositions during the whole sample period. Nonetheless, this balance displays considerable *ilr*-dispersion, which is reflected in the corresponding boxplot. Conversely, balances $b_6$-$b_9$ are characterized by low *ilr*-dispersion, with mean balances that are close to the center in *ilr*-8 and *ilr*-9, and are shifted right in *ilr*-6 and *ilr*-7.

**Fig. 10:**
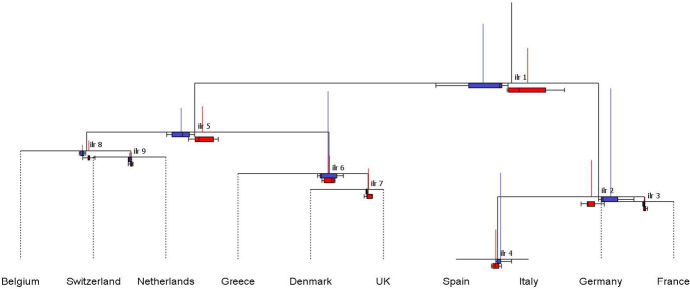
CoDa-dendrogram: balances of the SRISK composition with ten European countries according to the SBP in Table 6. In blue: first period (2008–2012), in red: second period (2013–2021) (color figure online)

The introduction of the temporal evolution factor in the CoDa-dendrogram, by splitting the considered years into two groups, (see Fig. 10) shows a considerable reduction in the variance of balances associated with the longest vertical bars ($b_1$, $b_2$, $b_4$, $b_6$), denoting a stabilization in SRISK shares after the GFC and the sovereign debt crisis. Interestingly, $b_1$ shows a considerable shift of the mean balance towards France–Germany–Italy–Spain, indicating an increase in the geometric mean of SRISK contributions for those countries relative to others. Inside the subcomposition, the influence of Italy and Spain has remarkably raised over time (cf. the results in Sect. 3.1). Balance $b_5$ shows a time decrease in the normalized logratio between Belgium–Netherlands–Switzerland and Greece–Denmark–UK, supporting the idea that the SRISK share of the former subcomposition has diminished over time relative to the latter. Balance $b_9$ stands out for its remarkable stability, with a mean located near the center of the horizontal bar. These features suggest that the (normalized) logratio between Netherlands and Switzerland is close to zero and approximately constant in both sample periods. Finally, a peculiar behavior is associated with balance $b_7$, which shows an increased contribution to the total variance in recent years. The associated boxplot indicates higher logratio dispersion between Denmark and UK.

### Inequality scenarios in SRISK compositions

In order to study the inequalities among SRISK shares pertaining to different European countries, we compute the Aitchison index (3) for each year in the sample period. For ease of interpretation, the measure is monotonically transformed into $A^2_{I,N} = 1 - \exp (A_I^2)$ and then expressed in percentage (cf. Egozcue and Pawlowsky-Glahn 2019). As shown in Table 7, inequality decreases sensibly in the period 2010–2012 (post-GFC), raises gradually between 2013 and 2017, and afterwards returns to lower levels.

Using the *ilr*-coordinates corresponding to the SBP partition in Table 6, the Aitchison index can be decomposed into balance components, as shown in the last term of equation (3). In order to visualize the compositional scenarios behind the dynamics of SRISK inequality during the period 2008–2021, Fig. 11 illustrates the individual contributions, *ilr*$_i({\textbf{x}})^2$, to the total value of $A_I^2$, with a focus on the first four *ilr*-coordinates. Interestingly, the balance between France–Germany–Italy–Spain and the rest of Europe (ilr.1, blue dotted line) has not contributed significantly to the inequality of SRISK compositions in 2008–2009, suggesting that the impact of the GFC has initially determined a similarity in the systemic weight of these two subsets of parts. However, the inequality contribution of ilr.1 becomes prevalent afterwards, indicating an increase in the relative importance of the first subset (France–Germany–Italy–Spain) with respect to the second (rest of Europe). The next component shows that the balance between France–Germany and Italy–Spain (ilr.2, orange dashed line) has played a major role in 2008–2009, but then has become progressively negligible. Indeed, the evolution of the corresponding SRISK parts (cf. Fig. 2, bottom panel) indicates that it has been a relative increase in the systemic risk share of Italy and Spain that has reduced the inequality contribution of this balance after the GFC. Also, the balance between Italy and Spain (ilr.4, green dash-dotted line) displays a relevant contribution to $A_I^2$ in 2009, but then tends to vanish as those countries have gradually reached a similar proportion in SRISK compositions, which appears to be permanently higher than their pre-crisis weight (cf. Fig. 1). Finally, the balance between France and Germany (ilr.3, gray dash-dotted line) is a minor contributor to SRISK inequality, notwithstanding a slight relative increase in the systemic weight of France in recent years.

**Table 7:** Aitchison inequality index for the SRISK composition of ten European countries during the period 2008–2021: the raw measure $A_I^2$, and the normalized version $A^2_{I,N}$ (percentage values)

Year	$A_I^2$	$A^2_{I,N}$ (%)
2008	1.2640	71.75
2009	1.5636	79.06
2010	0.8559	57.51
2011	0.8551	57.48
2012	0.7994	55.04
2013	0.9363	60.79
2014	1.0226	64.03
2015	1.2979	72.69
2016	1.4043	75.45
2017	1.4711	77.03
2018	0.8243	56.15
2019	0.8690	58.06
2020	0.9964	63.08
2021	0.8512	57.31

**Fig. 11:**
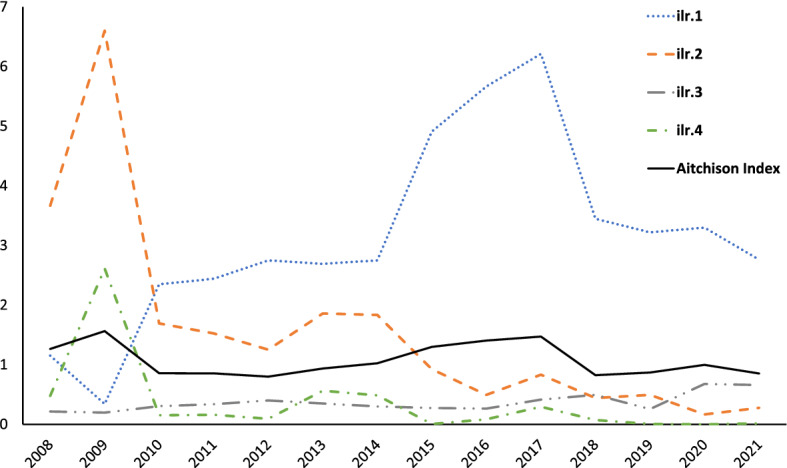
The Aitchison index $A_I^2$ (full line) and the contributions from some square balances associated to the *ilr*-coordinates in Table 6

## Discussion

In this paper we have performed an analysis of systemic risk in European financial institutions by the methodological tools provided by the compositional data approach. The considered dataset ranges between 2008 and 2021. The choice of 2008 as starting year has two motivations: the first one is technical, as the partitions related to the ten European countries in the previous years contain some zeros, making not applicable the compositional analyses, unless some zero-replacements (cf. Pawlowsky-Glahn et al. 2015). The second reason is instead related to the fact that the financial crisis consolidated in European Union from 2008, taking its time to cross the Atlantic Ocean, as exhaustively argued in Black et al. (2016).

The results described in the previous sections highlight the relevant role played by the first two components of the biplot in country clustering. As aforementioned, the first principal component groups Belgium, Netherlands, Switzerland and Germany, delineating a Continental region, which is opposed to a set of Euro-Mediterranean countries like Italy and Spain. The second principal component seems to be more related to the SRISK contribution of the countries, since it distinguishes the minor contributors (like Greece and Denmark) from the major ones (like France, Germany, Italy and UK). Thus the first principal component is possibly associated to a geographic feature, while the second one is related to financial characteristics. With respect to the second principal component, the locations of Greece and Germany show that they are the two ‘farthest’ countries in terms of SRISK contribution.

The comparison of rays and links in the covariance biplot allows to state that the most relevant country in terms of variability is Spain, meaning that its role has largely changed in the SRISK compositions over the observed time range. Even if the Spanish amount of SRISK is not among the largest ones, the CoDa analysis suggests that it can be considered a major driver of logratio variability for European SRISK compositions during the period 2008–2021, identifying it as source of potential instability for the whole European economy.

The results obtained from CoDa methodologies are to some extent consistent with previous research on the distribution of systemic risk in Europe and additionally lead to some novel findings that have not been explicitly observed with conventional analyses of absolute SRISK values. Firstly, the similarity of France and Germany as major contributors of systemic risk in the Euro area is clearly visible in the proximity of the corresponding vertices in the covariance *clr*-biplots. This behavior is a likely consequence of a well-established cooperation at all political, financial and economic levels, which makes the Franco-German relationship the driving force in the European Union. An interesting contribution that emerges from the SBP partition and the associated CoDa-dendrogram is that the SRISK shares of these countries relative to each other have remained essentially stable over time, with an *ilr*-balance steadily shifted towards France and very low *ilr*-dispersion. Thus, although the French banking system exceeds the German one in terms of absolute SRISK exposure (cf. Engle et al. 2014), their reciprocal SRISK proportions tend to maintain a certain equilibrium over time.

Secondly, the variation matrices and *clr*-biplots show that the most relevant contributions to the total variance of SRISK compositions can be attributed to the Euro-Mediterranean region (Greece, Italy, Spain). On the one hand, this result in line with a consolidated literature investigating the role of peripheral countries as determinants of systemic risk in Europe. In particular, MacDonald et al. (2015) have used the word “regionalism” to characterize the behavior of GIIPS economies (Greece, Ireland, Italy, Portugal, Spain), which have contributed and reacted to the spread of financial stress as a distinctive region. Furthermore, Black et al. (2016) have observed that “the interesting story leading into the sovereign crisis is the Italian and Spanish banks”, whose marginal contribution to systemic risk in Europe has grown significantly with the GFC, also in connection with contagion concerns flowing from Greece. On the other hand, the compositional perspective adopted in our paper sheds further light on specific differences among peripheral countries which are not easily captured by more traditional methods. As documented in an ample literature covering the pre- and post-crisis period in Europe (cf. MacDonald et al. 2015; Magkonis and Tsopanakis 2020), it is difficult to attribute a major role to Greece as a shock transmitter to the common currency area (at least in the degree that Italy is). As argued by González-Hermosillo and Johnson (2017), “one possible explanation for this is that Greece is seen as a country too small to affect Germany’s risk profile”, where the latter is regarded as a proxy for the resilience of the whole Euro area. Based on the analysis of financial stress indices, MacDonald et al. (2015) have documented a persistent role of Italy in stress transmission mechanisms among Eurozone economies, with some effects of Spain that have been mainly detected in the pre-crisis period. The relevance of Spain as a source of volatility in European Sovereign CDS markets has been considered in González-Hermosillo and Johnson (2017), while Magkonis and Tsopanakis (2020) have investigated the interconnectedness among the Spanish, Italian and German banking sectors and financial markets. Indeed, Greece and Spain are comparatively lower contributors than Italy in terms of absolute SRISK (cf. Fig. 1). However, an interesting finding that emerges from our study is that the percentage shares of Spain and Greece in SRISK compositions tend to exhibit higher dispersion (cf. Figs. 7, 9) and may consequently bring potential instability to the European financial system, even if their economies are comparatively smaller in size than other risk contributors. Compositional methods are particularly appropriate to detect this behavior because a relative scale can give better information than an absolute one when it comes to studying small proportions (see, e.g., Pawlowsky-Glahn et al. 2015).

A further contribution of CoDa methods concerns the relative comparison of SRISK shares among countries inside and outside the Eurozone. Following the orthogonality of some links in the covariance *clr*-biplot (Fig. 7), we have found nearly zero correlation between the logratios associated with UK–Denmark and France–Germany. This suggests that the SRISK balance between UK and Denmark (both non-Eurozone countries) tends to fluctuate differently than that between France and Germany (core Eurozone contributors), possibly as a consequence of diversity in the regulatory mechanisms operating at national levels. In this case, CoDa analysis appears to be consistent with some recent research based on absolute measures of systemic risk. In particular, Stolbov and Shchepeleva (2018) have argued that there may be no direct impact of the European Central Bank monetary policy on the systemic risk of countries which maintain national currencies other than the Euro; Dreyer et al. (2018) have outlined some unique features of the Danish banking system which may influence the country’s systemic risk exposure. Another interesting result emerging from CoDa-PCA is a close similarity between the SRISK parts pertaining to Switzerland and Netherlands, which are characterized by nearly proportional rays in the *clr*-biplot and a moderate logratio variability with a number of other countries. Although Switzerland and Netherlands have been usually regarded as relevant SRISK contributors in absolute values (see, e.g., Engle et al. 2014), the outcomes of CoDa analysis suggest that their SRISK proportions display a rather stable balance (cf. the CoDa-dendrograms in Figs. 9, 10) and are not strongly correlated with other parts in SRISK compositions (specifically, with Euro-Mediterranean countries like Spain and Greece, or else with UK and Denmark outside the Eurozone).

Additionally, the inclusion of a temporal evolution factor in CoDa-dendrograms (Figs. 6, 10) reveals that the total variance of SRISK compositions has significantly decreased after 2013. Several papers focusing on the impact of the financial and sovereign debt crises in Europe have indeed emphasized that, in absolute values, the systemic risk of European banks reached a peak between November 2011 and January 2012 (Black et al. 2016; Engle et al. 2014). As a complement to these studies, the CoDa analysis presented here indicates that, in the period following the European Banking Union and the reform of Basel Accords for the regulation of G-SIFIs, the distribution of SRISK shares among European countries has become less volatile in terms of relative proportions.

A major task attributed to SRISK (and, more generally, to a monetary index of systemic risk magnitude) concerns the evaluation of possible scenarios that could threaten the stability of the whole economy. These scenarios typically incorporate some risk concentration issues that depend on the relative proportions of SRISK parts in the system. Based on a CoDa measure of inequality and on its decomposition by balance-coordinates, we have been able to distinguish the contributions of specific subsets of European countries to the geographic distribution of SRISK shares over the period 2008–2021 (Fig. 11). In particular, the inequality contribution of the balance between France–Germany and Italy–Spain has been dominant in the GFC scenario, but has subsequently decreased as the SRISK share of Italy and Spain has progressively approached that of France and Germany. Conversely, the balance consisting of France, Germany, Italy and Spain over the rest of Europe has increasingly contributed to the inequality of SRISK proportions after the GFC, implying some dissimilarity in the post-crisis evolution of the former economies compared to the latter. This information is not easily detected in absolute values of a systemic risk indicator, and the proposed application of CoDa methods has proved useful in revealing an inequality dynamics inherent in alternative types of SRISK scenarios.

In light of these findings, our work underscores the importance of a continuous and accurate monitoring of systemic risk, suggesting the use of CoDa analysis in addition to traditional methods to enhance our understanding of how systemic risk originates and propagates across different countries. The conclusions of this study should be considered as part of a broader research area that tries to approach the complexity of systemic risk from multiple perspectives, and to propose appropriate tools for its detection and surveillance.

## Footnotes

1 Since PCA has a long standing tradition in multivariate statistics literature, we do not include here a detailed description. The interested reader can find a formal treatment of the methodology, for instance, in Chatfield and Collins (2018), and a characterization of PCA for CoDa analysis in the original work of Aitchison (1983).

2 In CoDa analysis, the concept of irregular data refers to the presence of zeros and/or missing values, which are not directly amenable to log-transformations (see, e.g., Egozcue and Pawlowsky-Glahn 2019; Pawlowsky-Glahn et al. 2015, among many others).

3 Including the third principal component would increase the percentage of total variance explained to $91.38\%$.
